# Physiological mechanisms of vascular response induced by shear stress and effect of exercise in systemic and placental circulation

**DOI:** 10.3389/fphar.2014.00209

**Published:** 2014-09-16

**Authors:** Iván Rodríguez, Marcelo González

**Affiliations:** ^1^Faculty of Health Science, Universidad San SebastiánConcepción, Chile; ^2^PhD Program in Medical Sciences, Faculty of Medicine, Universidad de La FronteraTemuco, Chile; ^3^Vascular Physiology Laboratory, Department of Physiology, Faculty of Biological Sciences, Universidad de ConcepciónConcepción, Chile; ^4^Group of Research and Innovation in Vascular HealthChillán, Chile

**Keywords:** endothelial dysfunction, shear stress, placental circulation, exercise, nitric oxide

## Abstract

Physiological vascular function regulation is essential for cardiovascular health and depends on adequate control of molecular mechanisms triggered by endothelial cells in response to mechanical and chemical stimuli induced by blood flow. Endothelial dysfunction is one of the main risk factors of cardiovascular pathology, where the imbalance between the synthesis of vasodilator and vasoconstrictor molecules is common in the development of vascular disorders in systemic and placental circulation. In the placenta, an organ without autonomic innervations, the local control of vascular tone is critical for maintenance of fetal growth and mechanisms that underlie shear stress response induced by blood flow are essential during pregnancy. In this field, shear stress induced by moderate exercise is one of the most important mechanisms to improve vascular function through nitric oxide synthesis and stimulation of mechanical response of endothelial cells triggered by ion channels, caveolae, endothelial NO synthase, and vascular endothelial growth factor, among others. The demand for oxygen and nutrients by tissues and organs, especially in placentation and pregnancy, determines blood flow parameters, and physiological adaptations of vascular beds for covering metabolic requirements. In this regard, moderate exercise versus sedentarism shows potential benefits for improving vascular function associated with the enhancement of molecular mechanisms induced by shear stress. In this review, we collect evidence about molecular bases of physiological response to shear stress in order to highlight the relevance of moderate exercise-training for vascular health in adult and fetal life.

## INTRODUCTION

The endothelium is the main regulator of vascular physiology, controlling hemodynamics and angiogenesis in postnatal and fetal life. Dysfunction of endothelial cells have several clinical implications related with alteration of physiological regulation of capillary permeability, vascular homeostasis, leukocyte trafficking, vasomotor control, angiogenesis, acquired and innate immunity, among others. Furthermore, these cells exhibit morphological and functional heterogeneity, which give them high capacity for adaptation, according to environmental conditions to maintain homeostasis in different vascular beds (Aird, 2007). In human placenta, an organ without autonomic innervations, the control of vascular tone is dependent on local release of vasoconstrictors and vasodilators, released from endothelial cells in response to mechanical and chemical stimuli triggered by cardiac output and blood flow requirements (Fox and Khong, 1990; Myatt, 1992). In placental and systemic circulation, the main stimulus regarding control of vascular resistance and blood flow, is related to increments of shear stress by high placental perfusion throughout pregnancy. The vascular response of placental circulation to shear stress depends of a variety of factors: local release of vasoactive molecules, endocrine signaling, oxidative stress in vascular cells or vascular remodeling, among others. The maintenance of vascular tone and blood supply for placental circulation is a key factor for adequate placentation and fetal development.

Relevant pathologies in developing and middle-income countries are NCDs, like CVD, metabolic syndrome, obesity and diabetes mellitus. With changes in lifestyle and quality of nutrition in western civilization, there is a sustained increase in DM2, GDM, overweightness and obesity during pregnancy (Kopp, 2005; Zhang and Ning, 2011; Pan et al., 2012). There is a consensus that NCD are serious problems for public health, and several public policies in the western world are oriented toward prevention based on healthy nutrition and exercise. For this reason, the aim of this review is to contribute to the understanding about molecular mechanisms involved in vascular dysfunction and deterioration of shear stress response, considering the positive effect of physical exercise on systemic and placental circulation. In this field, it is well known the importance of intrauterine life conditions for the development of NCD in adults (Hanson and Gluckman, 2011), so our focus is to show evidence about the effects of physical exercise and moderate training to better vascular adaptations triggered by shear stress, and the potential application of exercise therapy for improve systemic and placental vasculature function affected by chronic or gestational diseases.

## ENDOTHELIAL DYSFUNCTION

Endothelial dysfunction is established as one of the main risk factors for CVDs, where the imbalance between synthesis of vasodilators and vasoconstrictors is the most common factor (Qian and Chi, 2012). High synthesis and activity of vasoconstrictor molecules, such as endothelin-1 and thromboxane A2; as well as reduction in the availability of vasodilator molecules, like NO, associated with oxidative stress by high synthesis of ROS, are the cornerstone in poor capacity of endothelium to maintain vascular tone (Seals et al., 2011). Oxidative stress is a condition defined as an imbalance between systemic manifestation of ROS and a biological system’s ability to readily detoxify the reactive intermediates or to repair the resulting damage (Valko et al., 2007). In pregnancy, a condition associated with high risk of oxidative stress, the equilibrium between vasodilators versus vasoconstrictors and oxidative stress versus antioxidant mechanisms is a key factor during gestation and fetal development (Sobrevia and González, 2009).

In HUVECs from GDM or incubated with high concentration of D-glucose, there is high synthesis of NO (Sobrevia et al., 1995), related with high expression of the main L-arginine transporter: hCAT-1 (González et al., 2004, 2011; Guzmán-Gutiérrez et al., 2014). L-Arginine is the substrate for NO synthesis by eNOS (Alderton et al., 2003) and the transport of L-arginine is essential for NO synthesis in the endothelium (Shin et al., 2011). In assays where cells are incubated with high concentrations of D-glucose, the activation of the L-arginine/NO pathway has been related with endothelial dysfunction induced by concomitant increased synthesis of ROS and lower bioavailability of NO (Sobrevia and González, 2009). In this regards, a hallmark for endothelial function is the availability of NO, which is regulated by a combination of synthesis and NO inactivation (Luiking et al., 2010). Reduction in synthesis or availability of NO is associated with lower expression or activity of eNOS as a result of endogenous and exogenous inhibitors or by reduced availability of L-arginine (Dhein et al., 2003; Endemann and Schiffrin, 2004; Kalinowski and Malinski, 2004). Another important mechanism for NO inactivation and endothelial dysfunction is oxidative stress induced by ROS synthesis mediated by NADPH oxidases; enzymes whose primary function is to generate ROS and which plays an important role in redox signaling (Lambeth, 2004). The over expression or high activity of NADPH oxidase induces the uncoupling of eNOS due to the oxidative degradation of BH_4_, eNOS cofactor, leading to eNOS-dependent synthesis of superoxide anion (O_2_^-^) in detriment of NO synthesis (Antoniades et al., 2006; Dworakowski et al., 2008). Once synthesized, O_2_^-^ is used by SOD to generate H_2_O_2_, which has greater stability and capacity to diffuse through biological membranes, acting as a modulator of signal transduction pathways (Li and Shah, 2004). In addition, the O_2_^-^ reacts rapidly with NO to generate peroxynitrite (ONOO^-^), a powerful oxidizing agent that induces DNA fragmentation and lipid oxidation (Carr et al., 2000). Currently, it is postulated that the mechanism by which oxygen “hijack” the NO is related with the ONOO^-^ formation, which plays a central role in the development of endothelial dysfunction in diseases such as diabetes mellitus (Rolo and Palmeira, 2006; Hadi and Suwaidi, 2007; Rask-Madsen and King, 2008), preeclampsia (Gu et al., 2006; Escudero and Sobrevia, 2008), and hypertension (Harrison et al., 2003).

Also alterations related with endothelial dysfunction are associated with pro-thrombotic and pro-inflammatory states, and become the main etiologic factors for developing essential hypertension and atherosclerotic disease (Savoia et al., 2011). Determination of endothelial dysfunction in healthy and pathologic patients (especially during pregnancy) is a relevant challenge for physicians and researchers, regarding the obvious difficulties in extrapolating *in vitro* findings to the clinics. Therefore, non-invasive evaluation for endothelial dysfunction, such as FMV, are important tools to determine the association of endothelial dysfunction with wall thickness of conduit vessels, changes of pulse wave velocity and early cardiovascular risk predictors. In fact, these methods have been considered as complementary methods of the current evaluation guidelines for preventing CVD (Kozlov et al., 2012). Also the evaluation of endothelial dysfunction during pregnancy could be a potent tool in the prevention of CVD in early stages of development or in mothers that suffer pregnancy pathologies such as pregnant hypertension, preeclampsia, or GDM (Escudero and Sobrevia, 2008; Escudero et al., 2013).

## SHEAR STRESS IN SYSTEMIC AND PLACENTAL CIRCULATION

Shear stress is defined as the force exerted by the blood flow on blood vessel walls. This stress generates a response in the vascular wall, characterized by release of endothelial mediators, which in turn stimulate structural remodeling through activation of gene expression and protein synthesis (Hudlicka and Brown, 2009). Hemodynamic forces exerted by the heart during the cardiac cycle, PP and TS, change the structure of vascular wall. PP (difference between systolic and diastolic pressure) induces distention of the vascular wall which increases the radial tension on the blood vessels. TS or shear stress depends on the inner diameter of the vessel, blood flow rate, viscosity of the blood, and pulsatility of blood flow. It is estimated using Poiseuille’s law, through the product of shear on the wall and blood viscosity:

$$\tau =\frac{4*\eta *q}{\pi *r^{3}}$$

where η is fluid viscosity, *q* is flow, and *r* is radius. It is worth noting that this formula should be considered only for a blood vessel with circular cross section and in laminar flow regime. On the other hand, in clinical studies, shear stress is calculated through blood viscosity and shear rate (γ), which is estimated from the values of blood flow velocity (*V*) and internal arterial diameter (d) according to the following equation (Reneman et al., 2006):

$$\gamma =\frac{8*V}{d}$$

Shear stress values calculated in this way might be held for *in vitro* assays, provided that the conditions meet Poiseuille’s law. The latter statement cannot be applied to blood vessels *in vivo*, considering the presence of non-newtonian fluid, distensible vessels, pulsatile flow, and branching of the arterial tree. Moreover, blood flow velocity, and wall shear stress, is high in systole and relatively low in diastole. Thus, diastole comprises approximately two thirds of the cardiac cycle, and the level of wall shear stress during this phase of the cardiac cycle contributes substantially to the mean wall shear stress (Reneman et al., 2006).

In the case of placental shear stress, the same equations can be applied, considering that the placental flow is dependent on the umbilical blood flow, which is related with the umbilical vein diameter. In the placental vascular bed there are several hemodynamic adaptations in order to supply oxygen and nutrients to support the fetal growth. Endothelial cells are mainly responsible for these adaptations given that in the tunica intima where blood flow exerts longitudinal shearing forces (Sprague et al., 2010). Although there are obvious difficulties to determine changes in blood flow in fetuses during human pregnancy, some evidence obtained through non-invasive techniques like Doppler ultrasound has been used to determine the importance of placental vascular adaptation. To determine the umbilical blood flow in human pregnancy, Link et al. (2007) used this equation:

$$Q=V*d^{2}*\pi *0.15$$

where *Q* is the volume of umbilical blood flow (mL/min), *V* is the mean velocity (cm/s), and *d* is the diameter of umbilical vein (mm). In this study, the mean umbilical venous blood flow velocity was between 13 and 14 cm/s and was similar in preterm and full-term infants, whereas the diameter of the umbilical vein was greater in the full-term group. In preterm pregnancies, there was a decrease of umbilical blood flow in late pregnancy correlated with both gestational age and birth weight and the umbilical blood flow per unit body weight of the fetus or per placental weight was increased in preterm group. The authors argue that the increase of umbilical blood flow in the course of gestation is dependent of umbilical vein growth and there is a physiological decrease in the ratio between umbilical blood flow and fetal body weight that could be dangerous in post-term pregnancies (Link et al., 2007). These results show that the development and local regulation of umbilical vein diameter are determinants for an adequate blood flow to the fetus, considering that the endothelial cells respond to shear stress and there is no innervation in placental vasculature (Sprague et al., 2010). On the other hand, in isolated cotyledon from placenta, the increases of flow rate range from 1 to 10 ml/min increased the perfusion pressure, exhibiting a stronger effect when NO synthesis was inhibited (Wieczorek et al., 1995). Also, regulation of shear stress response in uterine vasculature is relevant for placental blood flow. For instance, in myometrial arteries from preeclamptic women there is no increase of flow rate by shear stress and lower capacity of induce NO-dependent relaxation. This might contribute to impaired utero-placental blood flow in this disease (Kublickiene et al., 2000).

Other important factors that regulate vascular response to shear stress are blood flow characteristics (magnitude and shape) and vascular tree anatomy (Friedman et al., 1987). For instance, it is well known that turbulence in zones of arterial branching, where oscillatory shear stress is generated, constitute areas of vascular remodeling associated with starting events leading to atherosclerosis (Giddens et al., 1993). It has been demonstrated that the flow patterns in ascending aorta contribute to pro-atherosclerotic environment, mainly that low and oscillator shear stress, specifically near of the aortic sinus. There is a correlation between low shear stress and increased incidence of vascular damage, especially near to the coronary arteries (Suo et al., 2008). Moreover, a study about structure and flow with 3D magnetic resonance in healthy subjects, established that the WTI is positively correlated with flow shear stress. Additionally, WTI is negatively correlated with atherosclerotic plaques wall stress, showing an increased progression of atherosclerotic plaques in zones of turbulent blood flow. This demonstrates that anatomic conformation of vascular beds and flow characteristics have important repercussions on endothelial damage development (Yang et al., 2010).

## MOLECULAR MECHANISMS INDUCED BY SHEAR STRESS

Mechano-transduction induced by shear stress is widely studied, showing that there are multiple signaling pathways which are activated in response to stress in endothelial cells (Li et al., 2005; Gautam et al., 2006; Yu et al., 2006; Jacob et al., 2007; Kumagai et al., 2009; Herranz et al., 2012). These pathways are triggered by mechanical stimuli sensed by endothelial cells, and generate intracellular signaling through second messengers, which in turn lead to the establishment of an adaptative response in short or long term according to stimulus (Johnson et al., 2011). For instance, the adaptive response of endothelial cells to the acute increase of shear stress is characterized by high endothelial cell permeability and high expression of anti-inflammatory and antioxidant proteins. This process is generated in three phases: induction, early adaptive response and late remodeling response, showing a different phenotype according the phase in which it is found (Zhang and Friedman, 2012).

### eNOS AND CAVEOLAE

Recently, a systematic review and 3-stage meta-analysis of studies that measured FMV under local infusion of saline or (L-NMMA; NOS inhibitor) solutions demonstrated that FMV of conduit arteries in humans is, at least in part, mediated by NO (Green et al., 2014). Furthermore, one of the enzymes that increases its expression in response to shear stress is NOS (Yee et al., 2008), specifically eNOS (Luiking et al., 2010). The use of NOS inhibitors, like L-NMMA or L-NAME, showed that the inhibition of NO synthesis suppresses the effect of shear stress on angiogenesis associated with muscular stimulation (Hudlicka et al., 2006) or placental microcirculation (Wieczorek et al., 1995). Still there is little evidence about the relevancy of L-arginine transporters in the response to shear stress. However, considering that NO synthesis depends on hCAT-1 activity (Shin et al., 2011), and has been demonstrated the colocalization of hCAT-1 with eNOS in caveolae (McDonald et al., 1997), it is highly probable that hCAT-1 is part of this physiological response. Importantly, the structure and function of caveolae is relevant for endothelial physiology: several studies have revealed that this subset of lipid structures, highly enriched in cholesterol and sphingolipids, play an important role in regulation of cell signaling (Das and Das, 2012; Sowa, 2012). Proteins such as cav 1, 2, 3 are part of their structure and organization, being cav-1 the more important in vascular endothelium (Hansen and Nichols, 2010). Together with cav-1, other proteins found in the caveolae are tyrosine-kinase receptors (TKRs), GPCRs, VEGFR, Ca^2+^ channels, among others. These expression profiles show the relevance of this plasma membrane structure for endothelial cells metabolism and vascular health (Sowa, 2012). It has been demonstrated in BAECs, that 1–3 days of exposition to laminar shear stress, increased the total amount of caveolae in 45–48%; as well as the expression of cav-1, compared with the same conditions without flow (Boyd et al., 2003). In cav-1 knockout animals (cav-1^-/-^) the decrease of shear stress for 14 days did not reduce the diameter of arterial lumen and exhibit high vascular wall thickness associated with reduction in the FMV and eNOS phosphorylation in serine 1176 (i.e., eNOS activation; Yu et al., 2006). Importantly, it has been observed that the association between cav-1 and eNOS is necessary for angiogenic response induced by shear stress, because cav-1 gene suppression decreases the response to VEGF stimulation, NO production and endothelial tube formation (Sonveaux et al., 2004). Like other endothelial cells, endothelial cells from the placenta and umbilical cord express cav-1. In oFPAEs, the effects of FGF-1 on proliferation and tube formation were abolished when stable cav-1 knockdown oFPAE was used (Feng et al., 2012). Also in HUVEC, the decrease of cav-1 suppressed the NO synthesis and tube formation induced by VEGF (Pan et al., 2006). Interestingly, in human and murine placenta there is a high expression of cav-1 and cav-2 in endothelium and VSMCs but there is a lack of expression in syncytiotrophoblast layer or in cytotrophoblast (Lyden et al., 2002; Mohanty et al., 2010). Although there is evidence that supports the role of cav-1 in placental vasculature, findings are lacking about specific effects of shear stress on co-localization of cav-1 or cav-2 with eNOS or hCAT-1 in human endothelium.

### ION CHANNELS

Another mechanism that is involved with the response to shear stress is related with the activity of ion channels in vasculature. Some ion channels activated by mechanical stress suffer conformational changes which modifies the cell membrane potential through changes of ions conductance (Sukharev and Sachs, 2012). Vascular endothelium expresses a great variety of sensitive channels for calcium (Ca^2+^), potassium (K^+^) and chloride (Cl^-^) ions, which elicit a rapid response of endothelial cells to shear stress (Nilius and Broogmans, 2001). In this context, K_ir_2.1 has shown to be a sensor of laminar flow, responding according to shear stress intensity in order to induce cell membrane hyperpolarization (Hoger et al., 2002). Together with K_ir_2.1, ORCCs are also activated simultaneously in presence of shear stress, whose stimulation induces endothelial cell membrane depolarization (Nilius and Broogmans, 2001). It has been demonstrated that chloride currents are saturated at 3.5 dyn/cm^2^, meanwhile K^+^ currents are saturated between 10 and 15 dyn/cm^2^. This shows that ORCC and K_ir_2.1 channels work in cooperation in order to provide sensibility to the endothelium for a wider range of shear stress. The Cl^-^ channel is responsible for sensing low levels of shear stress, and K^+^ channel is responsible for sensing high levels of laminar shear stress (Gautam et al., 2006). In this context, as membranes hyperpolarize during high shear stress, exercise-induced shear stress would be an important hyperpolarizing stimulus which would induce vascular relaxation of smooth muscle cells (SMCs; Gautam et al., 2006; Gurovic and Braith, 2012).

In placental tissues expression has been shown of K_V_, K_Ca_, K_ir_, and TASK1. Regarding function, NO-mediated relaxation of human umbilical arteries occurs via activation of K_V_ and K_Ca_ channels; K_IR_6.1 play an important role by reverse constriction in disease states, such as IUGR (Wareing et al., 2006). In the last decade it has been determined that insulin induces relaxation in umbilical and placental veins in a mechanism that could be dependent on activity of potassium channels (González et al., 2004, 2011). Specifically in HUVEC, the L-arginine transport and hyperpolarization induced by insulin is blocked by pre-incubation with glibenclamide, an inhibitor of K_ATP_ (González et al., 2004). Despite the importance of K^+^ channels in vascular response to shear stress and recent evidence about K^+^ channel expression and activity in human placenta, the role of K^+^ channels in placental shear stress and/or in complicated pregnancies is poorly understood (Wareing, 2014).

### VEGF AND ANGIOGENESIS

Modifications of blood flow induce changes in growth patterns of vascular beds, where an increase of the capillary/fiber ratio (C:F) in response to prolonged stimulation to shear stress and ischemic remodeling, decreases the diameter of capillaries and angiogenesis of low blood flow areas (Hudlicka and Brown, 2009) associated with VEGFR-2 (De la Paz et al., 2012). There are three receptors of VEGF (VEGFR 1, 2, and 3), being VEGFR-2 a strong tyrosine-kinase protein with high expression in vascular cells but reduced affinity to VEGF compared to VEGFR-1. Both receptors have soluble splicing isoforms, which contribute to negative regulation of angiogenesis. In this context, membrane-linked VEGFR-2 is pro-angiogenic, whereas sVEGFR1 or sFlt-1 is anti-angiogenic (Shibuya, 2013). Angiogenesis induced by shear stress is associated with NO bioavailability because the increase of collateral blood flow induced by VEGF and FGF is dependent on NOS activity (Yang et al., 2001). Also, L-arginine supplementation contributes to the increase in VEGF expression and angiogenesis in skeletal muscle and left ventricle of middle-aged rats, showing the importance of the L-arginine/NO pathway in VEGF expression in response to shear stress (Suzuki, 2006).

In placental circulation, it has been determined that the VEGF/angiogenesis pathway is relevant for early placental vascularization and deficiencies in this signaling pathway could be related with placental pathologies like IUGR or preeclampsia. Is well known that plasma levels of sFlt-1 is higher in mothers with preeclampsia (Shibuya, 2014) which is associated with lower NO synthesis in HUVEC obtained from mild or severe preeclampsia (Veas et al., 2011). Regarding placental responses to shear stress, these are similar to those reported in systemic circulation have been observed in oFPAEs, which shows high eNOS expression and rapid phosphorylation of eNOS on serine 1177 (Ser1177) through a PI3K-dependent pathway after applications of shear stress (Li et al., 2005).

## EFFECTS OF PHYSICAL EXERCISE ON SYSTEMIC SHEAR STRESS

Clinical evidence shows that physical exercise applied in cardiovascular rehabilitation is effective in decreasing both hospitalization rate and mortality associated with CVDs (Heran et al., 2011). In this regard, a study including 18.809 patients from 41 countries, showed that physical exercise decreases the risk of AMI in people who have a history of ACS (Chow et al., 2010). On the other hand, there are recent advances shows that controlled exercise in pregnant women with risk factors for CVD, like obesity or overweight, improve cardiovascular parameters (Seneviratne et al., 2014).

### IMPACT OF EXERCISE ON ENDOTHELIAL FUNCTION

Previously we mentioned that increases in shear stress causes the release of vasodilator substances from the endothelium and, consequently, FMV. FMV has been used as a parameter of endothelial function in clinical protocols and is the support of therapies for improving cardiovascular performance through shear stress induced by exercise (Inoue et al., 2008; Santos-García et al., 2011). When referring to the discussion about the effect of exercise on shear stress and vascular health, it is important to establish that there is a large variability of flow patterns in response to different types of exercise. For instance, in incremental exercise of the lower limbs, significant increases of blood flow peaks have been observed, associated with a biphasic increase of blood flow in the brachial artery due to anterograde and retrograde flow which is correlated positively with the intensity of workloads (Birk et al., 2012; Gurovic and Braith, 2012). This retrograde flow observed in the radial artery (and perhaps in other vessels) may be due to the redistribution or the influence of retrograde diastolic flow, which is associated with lower limb exercise in the upright position (Green et al., 2002a,b). Meanwhile exercise of upper limbs induces anterograde flow proportionally to the workload (Green et al., 2005). In the same way, Tinken et al. (2009) in compared the effects of blood flow modification and shear stress on FMV, reporting that when the anterograde flow was increased by 30 min, the FMV increased. Also, they observed that when the anterograde flow was decreased (through a brachial cuff), the elevation in FMV is blocked, suggesting that FMV is modulated by differences in the magnitude of anterograde flow and shear stress (Tinken et al., 2009). Furthermore, it has been observed that low retrograde flow predisposes to NO dependent endothelial dysfunction, because it generates an altered FMV response, which is a hallmark of endothelial dysfunction (Thijssen et al., 2009). Regarding the impact of exercise intensity on endothelial function, it has been shown that aerobic exercise of moderate intensity (50% VO_2_ max) increases the endothelium-dependent vasodilatation through stimulation of NO synthesis. Nonetheless, high intensity exercise could be an oxidative stress signal (Goto et al., 2003). Thus, these authors evaluated the response of brachial blood flow to different exercise intensities (25% VO_2_ max, 50% VO_2_ max, and 75% VO_2_ max) in healthy subjects and they demonstrated that exercise at 50% VO_2_ max induces vasodilatation through high bioavailability of NO, whereas high intensity exercise was associated with an increase in the production of ROS (Goto et al., 2007).

### IMPACT OF PHYSICAL TRAINING ON ENDOTHELIAL FUNCTION

Physiological bases concerning of physical training on endothelial function is related with the facts that increases of blood flow and shear stress affect the synthesis of NO (Naylor et al., 2011). In this context, it has been demonstrated in both animals and humans, that exposure to repetitive exercise carried out during a prolong period of time increases the bioavailability of endothelial NO, as well as vascular collateralization (Hambrecht et al., 2003; Heaps and Parker, 2011; Lee et al., 2011). Hambrecht et al. (2003) in a study using samples from patients with stable coronary disease and surgery of CABG scheduled, showed that a daily training program of 30-min with a cycle ergometer and 30-min with treadmill for 4 weeks before surgery, significantly increases the endothelium-dependent vasodilatation, the flow rate in response to acetylcholine (Ach) and the FMV in LIMA (Hambrecht et al., 2003). Furthermore, they also found that endothelial cells isolated from the LIMA of patients who were trained, exhibited a higher expression and activation of eNOS and PKB/Akt compared with untrained patients, showing that repetitive increases of shear stress, triggered by physical training, enhance the NO bioavailability (Hambrecht et al., 2003). Moreover, in relation to the time course of these vascular changes, it has been described that functional adaptations precede structural adaptations. Tinken et al. (2008) observed that 2–6 weeks of exercise-training triggered an increase in NO bioavailability due to an increase in exercise-induced shear stress. After 6 weeks, NO levels returned to baseline due the normalization of shear stress on the blood vessel wall associated by vascular remodeling and angiogenesis (Tinken et al., 2008, 2010). These results show that physical training with moderate intensity schemes would be useful tools to improve the outcomes of therapies and/or cardiovascular surgeries.

## EFFECTS OF PHYSICAL EXERCISE ON PLACENTAL VASCULAR FUNCTION

Even though research shows a high increase of overweight and obesity in world population, especially in pregnant women, there are few reports published regarding the effect of moderate physical exercise in pregnancy. However, effects have been demonstrated of exercise-training on pro-angiogenic molecules in pregnant animal models, showing evidences that exercise-based interventions would be effective in preventing the onset of preeclampsia. In this context, Gilbert et al. (2012b) showed that 6 weeks of training increases the levels of cytoprotective molecules such as heat shock proteins (HSPs) 27, 60, and 90 in placentas from trained rats compared with sedentary controls. The authors pointed out that small HSP are involved in cellular protection against oxidative stress and apoptosis, meanwhile larger HSP facilitate eNOS-mediated NO synthesis. In addition, it has been observed that exercise increases free VEGF, decreases sFlt-1 and increases endothelial cell tube formation *in vitro*. In addition, exercise augments endothelium-dependent vascular relaxation compared with non-exercise control rats (Gilbert et al., 2012a). These findings suggest that physical activity before and during pregnancy stimulate molecular pathways that may yield benefits with respect to placental and/or vascular function, since the HSP is associated with the VEGF overexpression which leads to development of angiogenesis (Gilbert et al., 2012a,b).

Moreover, exercise can mitigate hypertension-associated physiological consequences in pregnant rat models. It has been observed, in spontaneously hypertensive pregnant rats, that the exercise recovers the fetal weight decreased by hypertension. After an exercise-training program, the placentas of hypertensive rats had a higher number of blood vessels in relation to the sedentary control group (Abate et al., 2012). In addition, it has been observed in female Sprague Dawley rats exposed to 6 weeks of voluntary exercise, that the placental ischemia-induced hypertension was attenuated by exercise as well as the restoration of angiogenesis. These changes were associated with high sensitivity of Ach-induced vasodilation in mesenteric vessels of exercise-trained pregnant rats (Gilbert et al., 2012b). Apparently, these physiological effects induced by exercise could be transferred to the fetus, since there are evidences that physical training attenuates the detrimental effect of low-protein diet on fetal growth and development, glucose homeostasis, serum leptin concentration, and oxygen consumption in the offspring from exercised-trained mothers (Fidalgo et al., 2013). The underlying mechanism of these effects could be related to metabolic changes associated with long-term effects of perinatal physical training such as exercise-induced blood flow redistribution as well as increase of insulin-like growth factors, growth hormone, and leptin after training (Turgut et al., 2006; Amorim et al., 2009; de Mélo Montenegro et al., 2012; Fidalgo et al., 2013). However, more direct evidence is necessary to verify these hypotheses.

Regarding the effect of exercise-training in preeclampsia, it has been demonstrated in a mouse model of the disease (transgenic female mice over expressing human angiotensinogen, which develop preeclampsia when mated with males overexpressing human renin) that the exercise-training decreases the proteinuria, cardiac hypertrophy, and vascular reactivity of placental vessels. Also, it was observed that PlGF was normalized in trained transgenic mice (Falcao et al., 2010). Studies done on humans have shown that aerobic exercise is an effective tool in maternal weight gain and cardiovascular risk control during pregnancy (Clapp, 2008; Lamina and Agbanusi, 2013) and that physiological basis of preeclampsia are both vascular dysfunction and oxidative stress, which improve with exercise-training (Goto et al., 2007; Brown and Garovic, 2011). However, evidence related with the beneficial effect of exercise-training on vascular function and preeclampsia prevention in pregnant women is scarce (Yeo et al., 2000). In this context, Ramírez-Vélez et al. (2013) demonstrated that exercise-training during pregnancy led to a 2-fold increase in eNOS expression and 4-fold increase in NO production in placental cytosol, as well as, 6% decrease in O_2_^-^ level and 26% in H_2_O_2_ production rate in human placental mitochondria. The training program consisted in 32 sessions, each session included 30-min of aerobic circuit training accompanied by an audio music recording and instructions which guided the participants to exercise at each station for approximately 1 min per station in a circuit of 10 stations. In a similar way of previously reported data by Gilbert in rats, there is an increase in placental efficiency (fetal weight/placental weight) in exercised-trained pregnant women. These responses are triggered, presumably, by exercise-induced shear stress (Ramírez-Vélez et al., 2013). Additionally, it has been observed that 20 min of moderate-intensity cycle ergometry is effective for improving angiogenic markers: higher serum PlGF and lower sFlt-1 and sEng concentrations in late gestation (Weissgerber et al., 2010). Regarding the effect of exercise and changes of lifestyle for control or prevention of GDM, a recent publication concludes that there is no strong evidence to support the benefits of exercise on insulin resistance or glucose tolerance in these patients, although they are known the effects on vascular function, oxidative stress and insulin tolerance in humans (Weissgerber et al., 2010; Halperin and Feig, 2014; Rynders et al., 2014). Additionally, it has been described that insulin influences beneficial changes in insulin signaling and NO/ROS synthesis in GDM placenta (Sobrevia and González, 2009; Westermeier et al., 2011). Interestingly, there is a new protocol being applied in New Zealand called IMPROVE. In this study, pregnant women (aged 18–40 years) with a BMI 525 ≥ 25 kg/m2 and will be subjected to an exercise regime that consists of home-based stationary cycling. Researchers will collect anthropometric data of mothers and children together with different metabolic and cardiovascular parameters (Seneviratne et al., 2014). In the future, it will be very important to follow these and others trials outcomes, aiming to determine if exercise, its type and intensity, improves cardiovascular parameters with special interest in the mechanism induced by shear stress in placental circulation. In a recent report, the effect was determined of 20 min/day of treadmill exercise in pregnant sow, on vascular function of the femoral artery from porcine offspring at 3, 5, or 9 months of age. Although it was not possible to determine changes in endothelial function, overall, this report demonstrates, for the first time, that maternal exercise during pregnancy can induce long-term vascular programming in adult offspring. The authors determined a decreased of relaxation in response to sodium nitroprusside (NO donor) and high levels of the regulatory subunit of myosin phosphatase MYPT1 in femoral artery of porcine (3 months age) from exercised pregnant sows (Bahls et al., 2014). The main limitation of this study is the use of only the femoral artery for the determination of vascular reactivity and protein expression, in detriment of resistance vessels, or directly determine the changes in placental or uterine vascular beds from exercised mothers. Also for the determination of molecular changes in endothelial function, isolation of endothelial cells is necessary for *in vitro* determination of protein expression to avoid the overlap with protein profiles of SMC. Even so, this work opens new possibilities in the exploration of the effects of exercise during pregnancy, however, more evidence is necessary in order to clarify the subjacent mechanism that induce the cited beneficial effects. In this context, a previous study shows that exercise in late gestation increases the umbilical blood flow without changes in fetal weight, showing a potential effect of exercise on umbilical vein function that could be related with molecular mechanisms influenced by shear stress (Harris et al., 2013). These data suggest that the potential role of physical activity or exercise-training could be different in healthy and pathological pregnancy, where the exercise on the last (i.e., GDM, preeclampsia, hypertension, and obesity) has potential to improve fetoplacental function. In this regard, in a mouse model of preeclampsia superimposed on chronic hypertension induced by overexpression of renin–angiotensin system, exercise before and during pregnancy decreased mean arterial pressure and induced normalization of VEGF and sFlt-1 placental and plasma levels at the end of gestation. These findings show that preeclampsia is associated with an anti-angiogenic shift that is initiated by the placenta, and exercise-training restores angiogenic balance (Genest et al., 2013). This last idea supports the notion that exercise-training improves several parameters associated with the development of preeclampsia such as the risk of CVD, DM2, and improves the maternal health in patients at risk for preeclampsia (Genest et al., 2012).

Thus, mechanisms associated with benefits of exercise include: (1) VEGF-induced angiogenesis in early placentation due to short hypoxic events induced by redistribution of blood during exercise; (2) High levels of PlGF and reduction of circulating sFlt-1 and sEng in late gestation of exercised women; (3) High resistance against oxidative stress and lipid peroxidation, (4) Lower levels of endothelin-1 (vasoconstrictor) and high expression of eNOS and antioxidant enzymes, increasing the bioavailability of NO (Genest et al., 2012).

Even though studies are still scarce and controversial, new findings show the potential role of exercise-training on placental endothelial function. It is necessary to increase and improve experimental evidence, with carefully designed protocols on time and type of interventions, exploring molecular mechanisms in isolated endothelial cells and SMC from uterine (mother), placenta, umbilical cord (placental tissue), and systemic resistance vessels in offspring. The combination of animal models and human trials will give new insights about the benefits of exercise to avoid deleterious effects of pre-gestational and gestational diseases that are growing in the human population.

## PERSPECTIVES

Evidence shows that hemodynamic stimulus plays a crucial role in modulating synthesis and bioavailability of endothelial NO both *in vitro* and *in vivo*. This allows us to provide the experimental evidence that supports the positive effects of physiological shear stress and exercise-induced shear stress in systemic and placental circulation.

**FIGURE 1:**
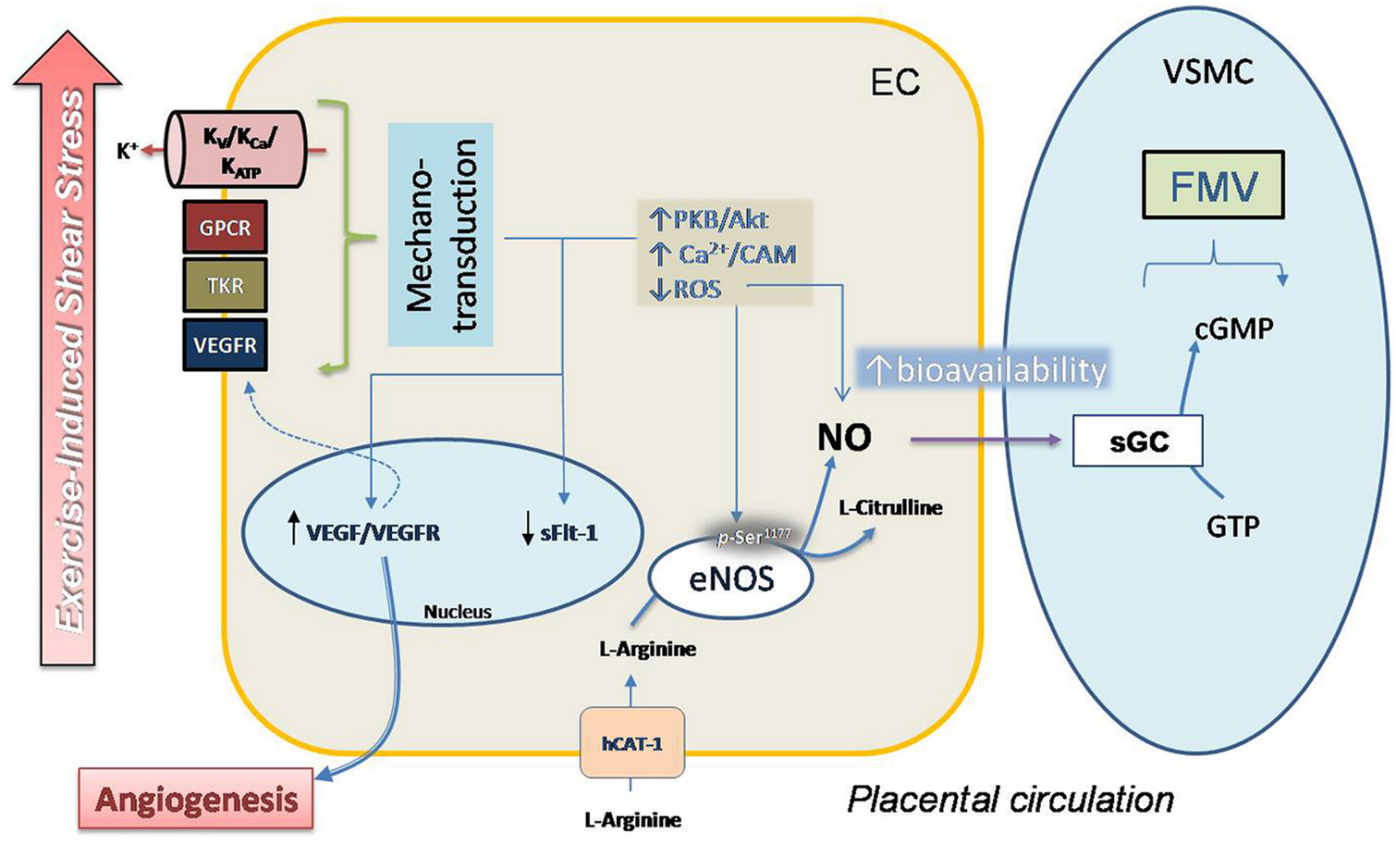
**Proposal model for regulation of FMV in placental vasculature in response to exercise.** Moderate exercise during pregnancy could improve the placental vascular response to shear stress in a mechanism that involves mechano-transduction induced by extracellular signals by a complex mechanism that includes activity of voltage (K_v_), ATP (K_ATP_) and K_Ca_, GPCRs, tyrosine-kinase receptors (TKRs) and VEGFR. Activation of this mechanism induces a signaling pathway that causes high phosphorylation (*p*-Ser1177) and activity in eNOS mediated by Ca^2+^/calmodulin (Ca^2+^/CAM) and protein kinase B (PKB/Akt). Decreased synthesis as well as reduced accumulation of ROS contributes to improve the bioavailability of NO. Activity of eNOS involves activity of hCAT-1, a protein co-expressed with cav-1 in caveolae (not shown), which is essential for NO synthesis. Diffusion of NO to smooth muscle cell (SMC) induces the activation of sGC and vascular smooth muscle relaxation.

The role of eNOS and VEGF in the regulation of physiological responses to shear stress is well known, and there is increasing evidence about the benefits of physical exercise (acute or training-exercise programs) for cardiovascular health. However, evidence about the effects of physical exercise on fetal and placental circulation remains obscure, mainly because of little information based on controlled trials in human pregnant women. In **Figure 1** we postulate a potential mechanism for response to exercise of placental vasculature, considering direct evidence and results obtained from systemic circulation and *in vitro* umbilical vasculature.

The endothelium is an inexhaustible source of cardioprotective substances which can be induced by exercise. For that reason, exercise-training can be considered an effective, economical and natural protector. Furthermore, light and moderate exercise does not have adverse effects, which cannot be said for the best medication synthesized in the pharmaceutical industry. These features, and the strong evidence about the high proportion of obesity and metabolic syndrome in pregnant women, demonstrate that the relevance of obtaining better knowledge about the effect of exercise-induced shear stress in fetoplacental vasculature.

## Conflict of Interest Statement

The Guest Associate Editor, Dr. Carlos Alonso Escudero, declares that despite having collaborated with author Marcelo Gonzalez in the past 2 years, there has been no conflict of interest during the review and handling of this manuscript. The authors declare that the research was conducted in the absence of any commercial or financial relationships that could be construed as a potential conflict of interest.

## ABBREVIATIONS

ACS: acute coronary syndrome
AMI: acute myocardial infarction
BAECs: bovine aortic endothelial cells
BH4: tetrahydrobiopterin
Cav: caveolin
CVDs: cardiovascular diseases
DM2: diabetes mellitus type 2
eNOS: endothelial NO synthase
FGF-1: fibroblast growth factor 1
FMV: flow mediated vasodilatation
GDM: gestational diabetes mellitus
GPCRs: G-protein couple receptors
hCAT-1: human cationic amino acid transporter 1
HUVECs: human umbilical vein endothelial cells
IMPROVE: ImprovingMaternal and ProgenyObesityVia Exercise
IUGR: intrauterine growth restriction
KATP: ATP-sensitivity K++ channels
KCa: calcium-activated K+ channels
Kir: inwardly rectifying potassium channel
KV: voltage-gatedK+ channels
L-NMMA: L-NGmonomethyl arginine;NADPH,nicotinamide adenine dinucleotide phosphate
NCDs: non-communicable diseases
NO: nitric oxide
oFPAEs: ovine fetoplacental artery endothelial cells
ORCCs: outwardly rectifying chloride channels
PlGF: placental growth factor
PP: pulse pressure
ROS: reactive oxygen species
sGC: soluble guanylate cyclase
SOD: superoxide dismutase
sVEGFR1: soluble VEGFR-1
TASK1: TWIK-related acid-sensitive K+ channels 1
TS: tangential stress
VEGF: vascular endothelial growth factor
VEGFR: vascular endothelial growth factor receptor
VSMCs: vascular smooth muscle cells
WTI: wall thickening increase.
